# Cronkhite-Canada syndrome presenting with chronic diarrhea: A case report

**DOI:** 10.1097/MD.0000000000045458

**Published:** 2025-10-31

**Authors:** Shenlu Wu, Junjun Wu, Yufang Wang

**Affiliations:** a The Second School of Clinical Medicine, Zhejiang Chinese Medical University, Hangzhou, Zhejiang Province, China; b Department of Gastroenterology, Hangzhou Third People’s Hospital, Hangzhou, Zhejiang Province, China.

**Keywords:** case report, Cronkhite-Canada syndrome, disease attributes, intestinal polyposis

## Abstract

**Rationale:**

Cronkhite-Canada syndrome, also known as gastrointestinal polyp pigmentation nail dystrophy/atrophy syndrome, is a rare, nongenetic disorder of unknown etiology characterized by gastrointestinal polyps and ectodermal abnormalities. Clinical manifestations commonly include abdominal pain, diarrhea, alopecia, skin hyperpigmentation, nail dystrophy, and dysgeusia. The syndrome may be associated with life-threatening complications, such as gastrointestinal bleeding, intussusception, recurrent pancreatitis, electrolyte disturbances, and hypoproteinemia.

**Patient concerns:**

This report presents a 66-year-old man admitted for recurrent abdominal pain and diarrhea. He was initially diagnosed with acute enteritis and treated empirically with antibiotics, which provided only transient relief. His symptoms recurred and progressively worsened >10 days after treatment cessation.

**Diagnoses:**

The patient had significant abdominal symptoms with ectodermal changes, and endoscopic and computed tomography computed tomography imaging of the small intestine revealed multiple polyps throughout the gastrointestinal tract (except the esophagus).

**Interventions:**

The patient was treated with glucocorticosteroids, mesalazine, proton pump inhibitors, rehydration with potassium-containing fluids, and nutritional support.

**Outcomes:**

After treatment with glucocorticoids combined with mesalazine, the abdominal pain and diarrhea improved significantly. However, due to poor compliance, the frequency of diarrhea increased to ten episodes per day after discharge. The patient was readmitted to the hospital due to a recurrence of symptoms and was treated with glucocorticoids and mesalazine. He was discharged from the hospital after his symptoms improved.

**Lessons:**

In this case, diffuse, variably sized, densely distributed, congestive polypoid changes were seen throughout the stomach, colon, rectum, and the terminal ileum. The lesions were more pronounced in the distal region of the gastric body than in the proximal region. Histopathology was suggestive of hamartomatous polyps with infiltration by eosinophils, lymphocytes, and plasma cells. Hormonal therapy has shown significant efficacy in treating this disease. Timely therapeutic intervention combined with systematic surveillance may mitigate the potential for malignant transformation and disease-related complications.

## 1. Introduction

Cronkhite-Canada syndrome (CCS) represents an uncommon acquired, nonhereditary form of gastrointestinal polyposis, typically manifesting with diffuse polyps throughout the digestive tract, accompanied by characteristic ectodermal abnormalities. The characteristic clinical triad consists of alopecia, cutaneous hyperpigmentation, and nail dystrophy affecting both fingers and toes; therefore, it is also known as the “polyp-pigmentation-alopecia-nail dystrophy syndrome.” It was first reported by Cronkhite and Canada in 1955 and was officially named by Jarnum and Jensen in 1966.^[1,2]^ The disease has a high mortality rate due to the multiple serious complications that may accompany it (e.g., cachexia, anemia, sepsis, and surgical complications). Data from a 1982 study showed that the 5-year mortality rate of patients with CCS was 55%.^[3]^ However, with the in-depth study of the disease and the improvement of treatment, a recent cohort study reported that the 5-year overall survival rate had improved to 87%.^[4]^ Currently, >500 cases have been documented worldwide.^[5]^ CCS is most commonly seen in the middle-aged and older age groups (mean age 59 years),^[6]^ and males are slightly more commonly affected than females. Studies in China and Japan have shown that the male-to-female ratio is 2:1 and 1.84:1,^[7,8]^ respectively. Endoscopically, CCS characteristically manifests as numerous congested polyps distributed diffusely throughout the gastrointestinal tract, with or without involvement of the esophagus.^[9]^ Histologically, the most characteristic histopathological feature of CCS is the presence of hamartomatous polyps, accompanied by lamina propria and submucosal congestion, as well as chronic inflammatory infiltration.^[8]^ Given that the pathophysiological mechanisms of CCS have not been fully elucidated, no standardized treatment protocols have been established.

The diagnostic evaluation of CCS primarily depends on characteristic clinical manifestations, endoscopic features, radiographic imaging, and histopathologic evaluation. To clarify the diagnosis of the current patient, further investigations, including histologic analysis of polyp specimens, were performed to confirm CCS from a pathologic point of view.

Finally, we systematically analyzed published CCS research to identify diagnostic and therapeutic advances with potential clinical applicability.

## 2. Case report

### 2.1. Case presentations

A 66-year-old male patient was admitted to the hospital with recurrent diarrhea, loss of appetite, abnormal taste, weight loss, nail loss, and abnormal skin pigmentation. He had been diagnosed with acute enteritis, which was relieved by antibiotic treatment but was recurrent, and the number of bowel movements per day ranged from 4 to >10. His past medical history included a history of pancreatic head, pancreatic body, and gallbladder resection, hypertension, and diabetes mellitus. His social history included a history of alcohol consumption for >30 years, with consumption of half a pound of white or yellow wine daily; however, he had been abstinent for 1 month. There was no significant family history.

**Figure 1.**
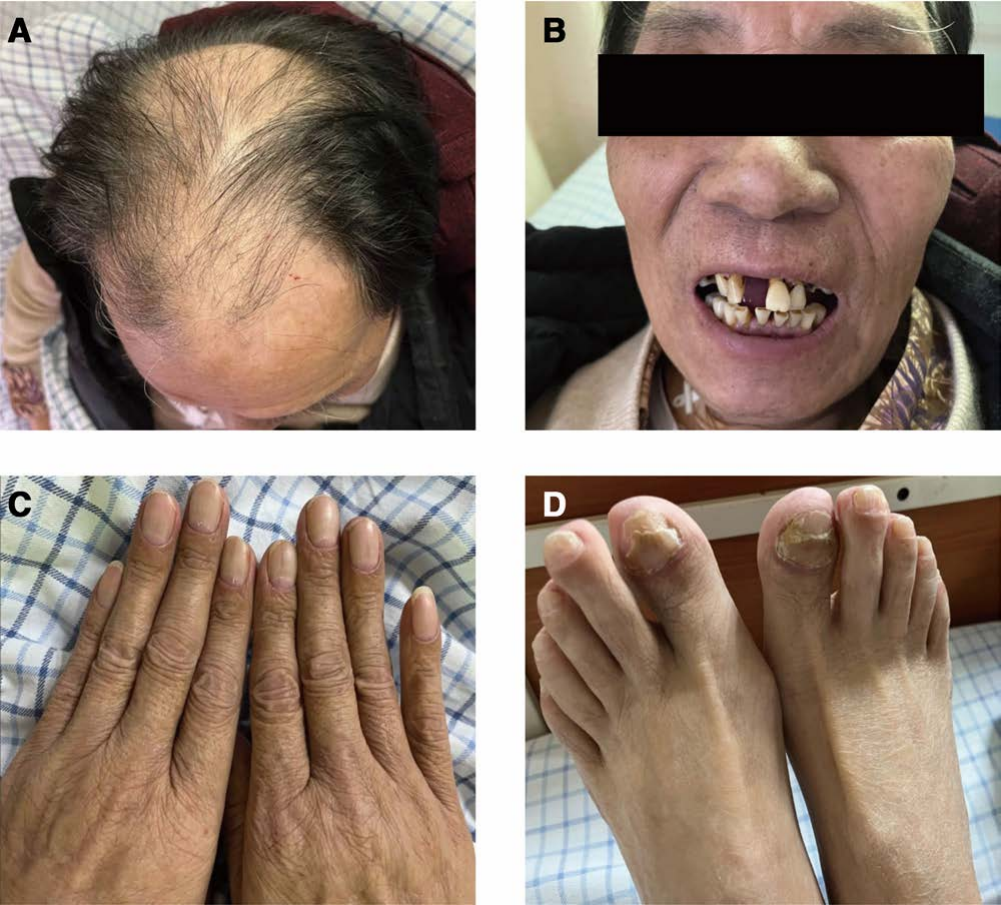
(A) Hair loss. (B) Facial hyperpigmentation. (C) Hyperpigmentation of the hands and rough nails. (D) Partial loss of toenails.

Physical examination revealed a long and thin body type, a height of 171 cm, and a weight of 64 kg. Skin pigmentation was present on both upper limbs, some toenails were peeling, and his hair was thinning and falling out (Fig. 1).

### 2.2. Laboratory tests

Laboratory examination: leucocyte count 6.2 × 10^9^/L, hemoglobin 127 g/L↓, IgG4 2.51 g/L↑ (0.030–2.010), cANCA (−), pANCA (−),antinuclear antibody (ANA −),PR3 (−),MPO (−), IgA 1.57 g/L (1.00–4.20), IgG 6.42 g/L↓ (8.6–17.4 g/L), IgM 0.18 g/L↓ (0.50–2.80), potassium 2.61 mmol/L↓ (3.50–5.30), calcium 1.75 mmol/L↓ (2.11–2.52), phosphorus 0.70 mmol/L↓ (0.85–1.51), magnesium 0.73 mmol/L↓ (0.75–1.02), prealbumin 111 mg/L↓ (150–380), total protein 39.3 g/L↓ (65.0–85.0), albumin 20.9 g/L↓ (40.0–55.0), total cholesterol 2.05 mmol/L↓ (2.84–5.19), triglyceride 0.94 mmol/L (0.34–1.69), low density lipoprotein 0.67 mmol/L↓ (1.55–3.39), D-dimer 0.85 mg/L↑ (0.00–0.55), TSH 1.005 mIU/L (0.380–4.340), blood sedimentation 1 mm/h, Cytokeratin 19 fragment 9.69 ng/mL↑ (0.00–3.60), CEA 3.09 ng/mL (0.00–5.00), HbA1c 5.7 % (4.6–6.5), fecal occult blood (+), fecal third line culture (vibrio cholerae, *salmonella*, and shigella), fecal Candida culture (−) and *Staphylococcus aureus* cultures (−).

### 2.3. Radiographic examination

**Figure 2.**
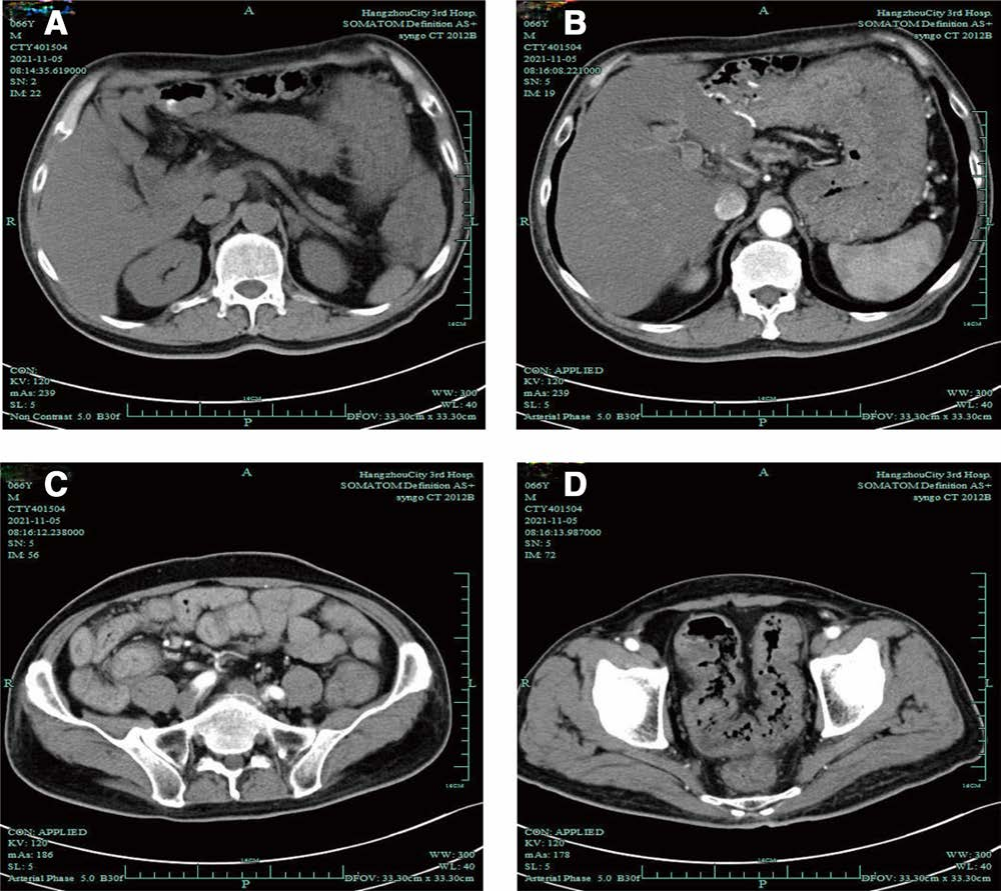
(A–D) Enhanced CT of the whole abdomen suggested more colonic contents, extensive thickening of the intestinal wall, obvious enhancement of the mucosa after enhancement, part of the intestinal lumen was narrowed, and the peripheral fat interstitial exudation was not obvious, and no enlarged lymph node shadow was seen. After gallbladder surgery; after pancreatic head and neck resection. CT = computed tomography.

Enhanced computed tomography (CT) of the entire abdomen suggested extensive thickening of the intestinal wall, multiple nodular soft tissue density shadows in the colon, and partial narrowing of the intestinal lumen (Fig. 2).

### 2.4. Endoscopic manifestations

**Figure 3.**
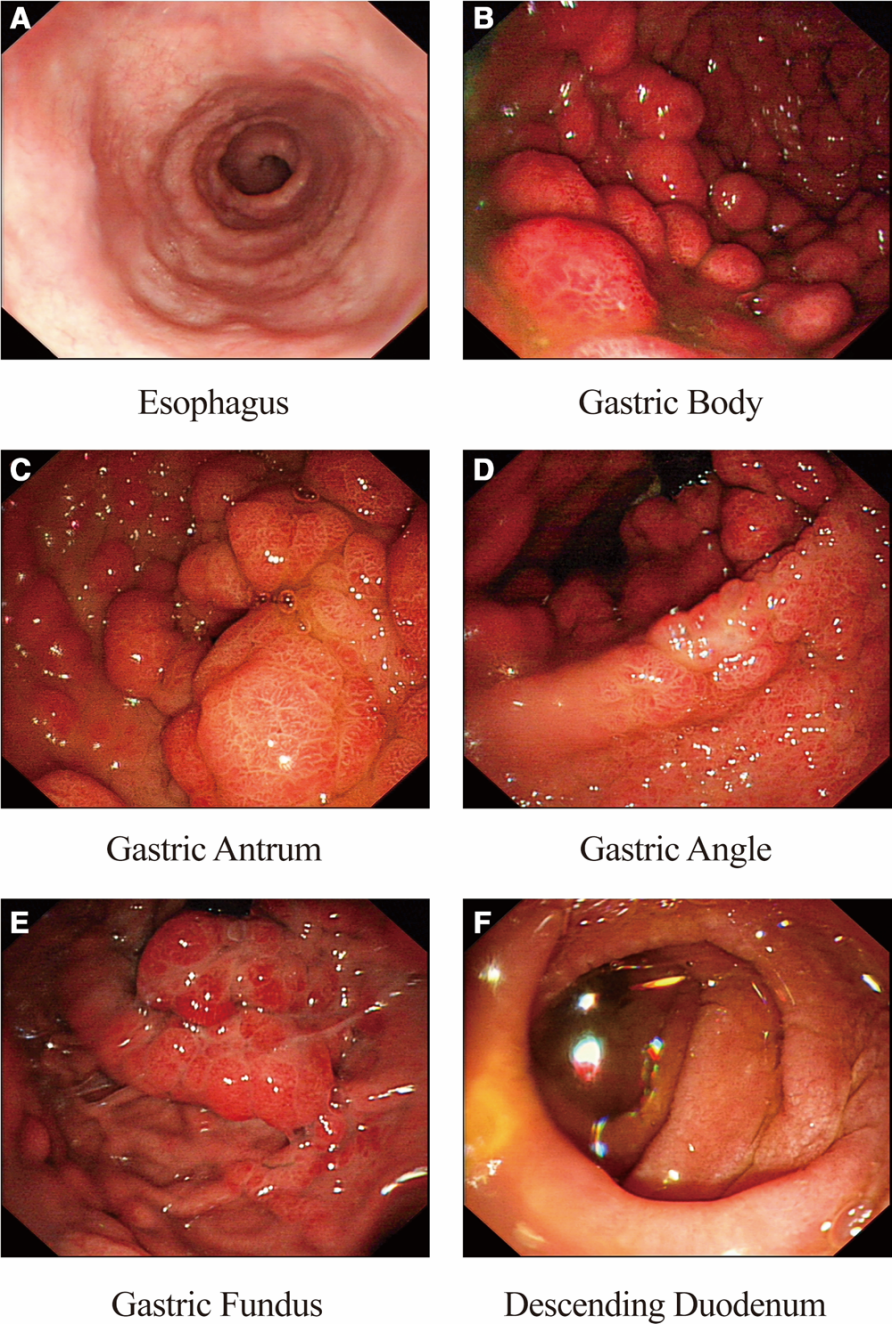
(A) The esophageal mucosa is smooth with well-defined vascular texture. (B–F) Diffuse, nodular and granular elevations of varying sizes gradually appeared in the gastric mucosa. The surface was smooth, reddish, and partially fused. The mucosa showed marked congestion and edema. (G) The duodenal mucosa is edematous, with no obvious polypoid changes.

Gastroscopy showed smooth esophageal mucosa with clear vascular texture. Diffuse polypoid elevations of varying sizes were seen throughout the stomach, with smooth, reddish surfaces, partly resembling a strawberry-like appearance. The lesions ranged from 0.2 to 2 cm in diameter, with the mucosa showing congestion and edema. The distal gastric body lesions were more prominent than the proximal ones, and the duodenal mucosa was edematous (Fig. 3).

**Figure 4.**
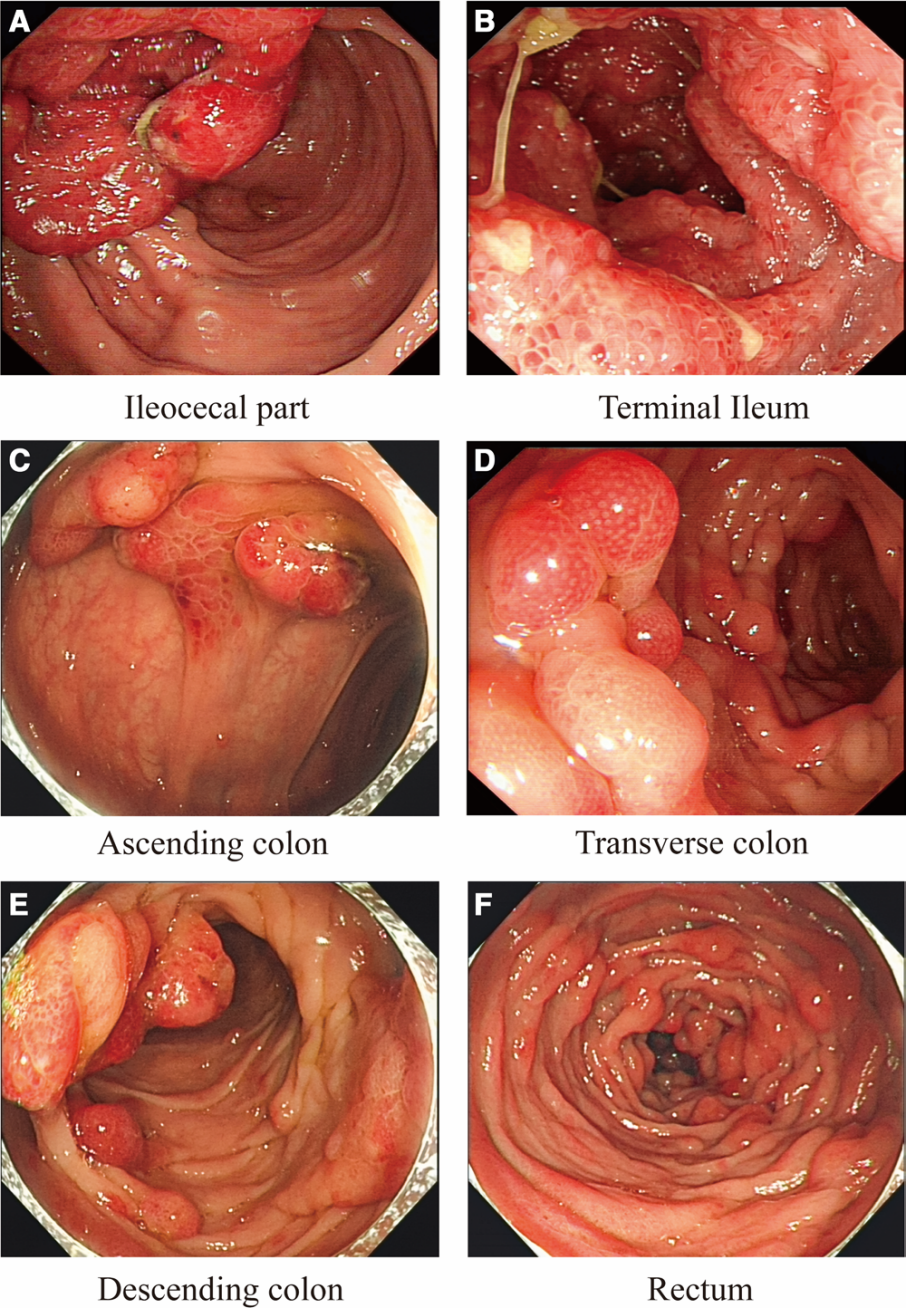
(A) Congested and swollen mucosa at the ileocecal valve is seen. (B) Multiple broad-based polypoid elevations of soft texture with mucosal congestion and edema are seen at the end of the ileum. (C–F) The entire colon and rectum show multiple broad-based polypoid and granular elevations of varying sizes with reddened surfaces and necrotic erosions at the tips of some polyps. The mucosa is congested and swollen.

Colonoscopic findings included diffusely distributed polypoid elevations of diverse dimensions (0.6–3 cm), characterized by erythematous, sessile, oval morphologies, with certain lesions presenting a grape-like cluster pattern. The heads of the polyps were congested and edematous (Fig. 4).

### 2.5. Histopathological examination

Gastric: moderate chronic inflammation (active) of the mucosa with localized mucosal erosions, glandular hyperplasia, edema of the lamina propria, more inflammatory cell infiltration, and localized lymphoid tissue hyperplasia.

**Figure 5.**
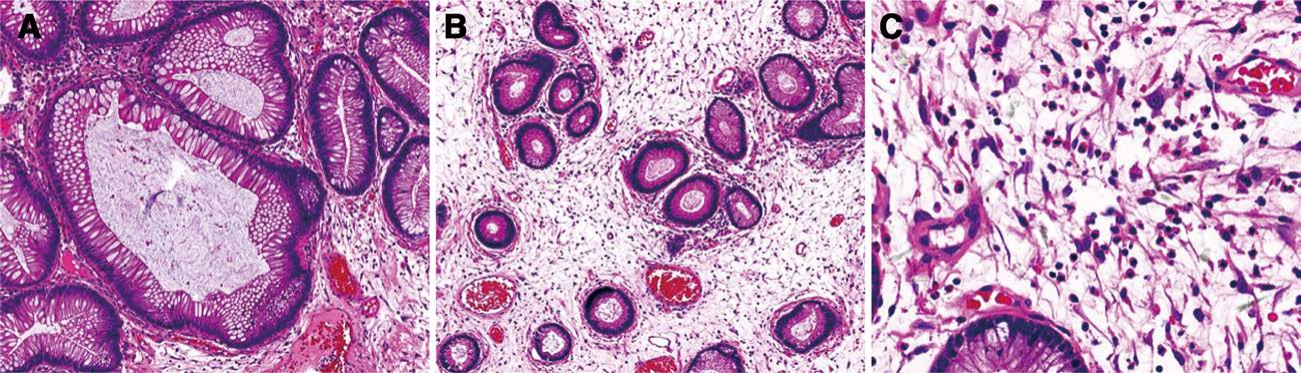
Histopathological examination of transverse colon polyp: (A) (HE× 100) Mature differentiation of glandular epithelium, with cystic dilatation of some glands, filled with mucus. (B) (HE× 100) Edema of the lamina propria of the transverse colon mucosa, with vascular hyperplasia and dilatation. (C) (HE× 400) Obvious inflammatory cell infiltration with eosinophils predominantly, accompanied by lymphocytes and plasma cells. HE = hematoxylin and eosin staining.

Colon and rectum: chronic inflammation (active) of the mucosa with mucosal erosions, cystic dilatation of some glands, edema of the lamina propria of the mucosa, predominantly eosinophilic infiltration in the interstitium, and lymphocytic and plasma cell infiltration (Fig. 5).

### 2.6. Diagnosis and treatment

In this case report, the patient’s earlier complete blood count did not show an elevated C-reactive protein level. However, an increased white blood cell count, inflammatory changes throughout the intestinal lumen observed on abdominal CT, and the patient’s history of diabetes mellitus collectively suggested that the chronic diarrhea might have led to gut microbiota translocation, defects in the intestinal mucosa, and mucosal congestion with edema. Consequently, the patient was diagnosed with acute enteritis and treated with antibiotics (levofloxacin, 0.5 g once daily), which yielded unsatisfactory results. Given that the patient had previously undergone pancreatic head and body resection due to a space-occupying pancreatic lesion, the recurrent diarrhea was considered possibly related to pancreatic exocrine insufficiency. Therefore, enteric-coated pancreatic enzyme capsules were administered to aid digestion and supplement enzyme levels. The patient had a long-term history of alcohol consumption, primarily of hard liquor and rice wine. CT angiography did not reveal significant sclerotic changes in the mesenteric vessels; thus, mesenteric venulosclerosis was deemed unlikely. However, the possibility of alcohol-induced chemical damage to the intestinal wall could not be entirely ruled out. Over the past year, the patient experienced a weight loss of approximately 10 kg. Although the patient had diabetes, and it was unclear if he usually had good glycemic control, diabetes could not be excluded as a potential contributing factor to the weight loss. The final diagnosis of CCS was established based on a comprehensive evaluation of the medical history and auxiliary examinations: the patient was a middle-aged male with no relevant family history; clinical manifestations included persistent diarrhea, alopecia, onychomadesis of the fingernails and toenails, and skin hyperpigmentation; endoscopic examination revealed multiple nodular polyps in the stomach and colon, with no esophageal involvement; pathological examination of the polyps revealed edema in the lamina propria, cystic dilation of some glands filled with mucus, and significant inflammatory cell infiltration predominantly composed of eosinophils, accompanied by lymphocytes and plasma cells, confirming the presence of both hamartomatous and adenomatous polyps.

The patient was administered methylprednisolone tablets 32 mg/qd, mesalazine 1 g/qid, pantoprazole sodium 40 mg/bid, calcium carbonate D3 0.75 g/qd, alaninyl glutamine injection 10 g/bid, pancreatic enzyme enteric capsules 300 mg/tid, rehydration with potassium-containing fluids, nutritional support, and other treatments. After 5 days of treatment, the frequency of diarrhea reduced from ten times per day to 3 times per day; therefore, the patient was discharged from the hospital. However, due to poor compliance, he discontinued the medications on his own. Half a month later, the frequency of diarrhea increased to >10 times per day, the patient was readmitted to the hospital, and he was readministered the above medications for 10 days. Afterwards, the frequency of diarrhea reduced to zero times per day, and the patient was discharged from the hospital.

### 2.7. Follow-up

During the 1-month post-discharge telephone follow-up, the patient reported no significant discomfort. However, the patient self-discontinued medication after 2 months and was lost to follow-up at the 8-month timepoint.

## 3. Discussion

### 3.1. Clinical manifestations and complications

CCS is a rare disorder that manifests with gastrointestinal polyposis, digestive symptoms, and ectodermal abnormalities. Gastrointestinal symptoms typically include abdominal pain and diarrhea, while ectodermal changes manifest as alopecia, nail dystrophy, and skin hyperpigmentation. Alopecia may involve hair on the head, eyebrows, and eyelashes,^[10]^ and and nail pathology shows increased matrix granules,^[11]^ indicating an inflammatory rather than a nutritional cause. In addition, patients with CCS may develop serious complications such as gastrointestinal bleeding, intussusception, duodenal papillary prolapse, recurrent pancreatitis, chronic inflammatory anemia, electrolyte abnormalities, hypoproteinemia, and other nutritional deficiencies caused by malabsorption. These patients are also at high risk of infection and have a 10% to 20% risk of gastric and other gastrointestinal cancers.^[5]^ In this case, the patient exhibited the typical clinical manifestations and complications of CCS, including diarrhea, abdominal pain, alopecia, partial loss of toenails, and facial hyperpigmentation, as well as gastrointestinal bleeding, electrolyte abnormalities, hypoproteinemia, and infections. The presence of these clinical manifestations and complications further supported the diagnosis of CCS.

### 3.2. Pathogenesis

To date, the pathogenesis of CCS remains incompletely understood. Studies have suggested that it may be associated with a variety of factors. Autoimmune factors: IgG4-positive plasma cell infiltrates in pathologic samples of polyps from patients with CCS,^[12,13]^ as well as antinuclear antibody positivity in some patients,^[14]^ suggest that an autoimmune response is involved in the pathogenesis of CCS. Infectious factors: some patients with CCS experience symptomatic relief after *Helicobacter pylori* eradication.^[15]^ In addition, Clostridium difficile and Klebsiella pneumoniae infections may contribute to the development of CCS.^[16,17]^ Stress factors: mental and physical stress have been identified as significant risk factors for CCS.^[18,19]^ Gene Mutational factors: genetic analysis suggests that mutations in the PRKDC gene may be involved in the pathogenesis of CCS,^[20]^ while aberrant expression of the inhibin βA gene may also be part of the pathogenesis of CCS.^[21]^ Other factors: these include thyroxine,^[22]^ traditional Chinese medicine, and hair dyes,^[5,14]^ which may affect the pathogenesis of CCS through different mechanisms. A study observed that CCS patient-derived organoids exhibit hyperproliferation and increased serotonin-producing enteroendocrine cells. Serotonin promotes epithelial proliferation, and its inhibition reverses this phenotype, revealing a mechanistic link and potential therapeutic target for CCS.^[23]^ Research has identified 543 dysregulated genes in CCS colonic polyps, which are related to innate immunity, extracellular matrix disorganization, inflammation, angiogenesis, and epithelial-mesenchymal transition. Quantitative real-time polymerase chain reaction validated key gene overexpression, supporting the role of chronic inflammation and highlighting the therapeutic potential of immune modulation and barrier repair.^[24]^ In this case, the patient’s morbidity may have been associated with *H pylori* infection, but the improvement of the patient’s symptoms after *H pylori* eradication could not be assessed because of his poor compliance and failure to undergo subsequent debridement therapy.

### 3.3. Complementary checkup

#### 3.3.1. Laboratory tests

Laboratory tests in CCS may reveal the following findings: decreased hemoglobin (which may be associated with chronic inflammatory anemia due to the patient’s chronic diarrhea and gastrointestinal bleeding), decreased albumin (which may be associated with gastrointestinal mucosal injury and dysfunction), electrolyte abnormalities (which may be associated with chronic diarrhea-induced electrolyte loss and malabsorption), and a positive fecal occult blood test (which may be associated with gastrointestinal mucosal injury). Some patients may also have the following findings: positive serum ANA, positive serum IgG4 test, positive *H pylori* test, and increased eosinophil count or elevated ratio in the peripheral blood. In this case, the patient’s laboratory findings were suggestive of decreased hemoglobin, decreased albumin, electrolyte abnormalities (low potassium, low calcium, and low phosphorus), positive fecal occult blood test, positive serum IgG4 test, and positive *H pylori* test. These results provided a crucial basis for diagnosis and treatment.

#### 3.3.2. Endoscopic manifestations

Endoscopic evaluation in CCS shows a multifocal distribution of polyps, predominantly in the stomach and colon (60–80% of cases), followed by the small intestine and rectum,^[12]^ which can involve the entire gastrointestinal tract. In a retrospective analysis of Japanese and Chinese patients with CCS, there was esophageal involvement in 12.3% and 3.88% of cases,^[8,25]^ respectively. Endoscopically, polyps usually appear as multiple, nodular, broad-based masses that range from 2 to 40 mm in size, and they may be accompanied by mucosal edema, congestion, hemorrhage, or erosion.

Within the stomach, polyps occur most frequently in the antrum, while pathological changes tend to be more marked in the distal portion of the gastric body than in the proximal segment.^[26]^ The small intestine tends to have sparsely distributed small polyps (<10 mm). Endoscopic examination of a patient with CCS without polyps revealed gastric mucosal edema, hyperemia, and mosaic pattern alterations, along with duodenal fold abnormalities and small intestinal nodular changes.^[27]^ Endoscopy showed no esophageal involvement in this patient, and wide basal nodular polyps of varying sizes were diffusely distributed throughout the stomach and colon.

#### 3.3.3. Histopathologic analysis

There is a wide variety of CCS-associated polyps, including inflammatory, hyperplastic, hamartomatous, and adenomatous polyps. Hamartomatous polyps are the most representative.^[28]^ The pathology of CCS polyps is characterized by a pronounced inflammatory cell infiltrate, mainly involving eosinophils, lymphocytes, and neutrophils.^[29]^ In addition, structural abnormalities of the glands or crypts (e.g., cystic dilatation, some with proteinaceous fluid or mucus retention), edema of the lamina propria, as well as edema, congestion, and inflammatory glandular hyperplasia of the inter-polyp mucosa are seen.^[3]^ Several investigations have demonstrated the presence of IgG4-positive plasma cells within CCS-associated polyps.^[12,30]^ Pathologic examination in our case revealed hamartomatous and adenomatous polyps, with infiltration of lymphocytes, eosinophils, and other inflammatory cells.

### 3.4. Diagnosis

The diagnosis of CCS lacks uniform criteria and usually relies on a comprehensive evaluation of the patient’s clinical history, physical examination, endoscopy, and pathologic findings. Goto categorized CCS into 5 clinical subtypes based on its first symptoms and pathogenesis^[18]^:

Type I (diarrhea-dominant type): with persistent diarrhea as the main manifestation; type II (dysgeusia type): characterized prominently by taste abnormalities; type III (dry mouth type): with significant dry mouth as the clinical hallmark; type IV (abdominal symptoms type): presents with abdominal symptoms other than diarrhea (e.g., chronic heartburn and nonspecific abdominal pain); type V (alopecia areata): characterized by hair loss throughout the body, including scalp hair, beard, eyebrows, eyelashes, axillary hair, and pubic hair, and accompanied by atrophy of the finger (toe) nails. In this case, the patient exhibited symptoms such as diarrhea, hair loss, and toenail atrophy, which can be categorized as type I and type V.

### 3.5. Differential diagnosis

Accurate clinical differentiation is required between gastrointestinal polyps and other polyposis syndromes, including Peutz-Jeghers syndrome, familial adenomatous polyposis, juvenile polyposis syndrome, and Cowden syndrome (Table 1). This requires considering the patient’s family history, the distribution and morphological characteristics of the polyps, accompanying symptoms, and endoscopic and pathologic findings. With comprehensive information, these diseases can be more accurately differentiated to provide targeted treatment programs for patients.

**Table 1:** Differential diagnosis of multiple polyps in the digestive tract.

	Cronkhite-Canada syndrome	Peutz-Jeghers syndrome	Familial adenomatous polyposis	Juvenile polyposis syndrome	Cowden syndrome
Age of prevalence	Middle-aged, elderly	Youth	Youth	Children	Youth
Genetic factor	Nonhereditary disease	Autosomal dominant	Autosomal dominant	Autosomal dominant	Autosomal dominant
Favored location	Stomach, colon, followed by small intestine	Jejunum, colon	Colon, rectum	Rectum, distal colon, stomach	Sigmoid colon, rectum
Extracolonic lesions	Taste disorders, hair loss, yellowing/shrinkage/loss of finger (toe) nails, skin pigmentation, etc	Pigmented spots on lips, mouth and skin	Congenital hypertrophy of the retinal pigment epithelium, epidermoid cysts, osteoma, desmoid tumor, hepatoblastoma, supernumerary teeth, thyroid cancer, brain tumor	Skin manifestations (e.g., dilated capillaries, pigmented nevi) and skeletal features (e.g., macrocephaly, hydrocephalus, cleft palate, polydactyly, wide eye spacing)	With facial papules, oral mucosal papillomas, and keratosis pilaris of the extremities
Endoscopic performance	Multiple, nodular polyps, often non-tipped or sub-tipped, about 2–40 mm in size	Polyps vary in size, most are <1 cm	The distribution of polyps in the colon is characterized by a diffuse and dense pattern, most of which are within 1 cm and are sessile or semipedunculated	Polyps are numerous and large in size, varying in size and number, and presenting as spherical polyps that are stalked(90.5%) or sessile(9.5%)	Size ranges from several millimeters to several centimeters
Pathological findings	Hamartomatous polyps, inflammatory polyps, hyperplastic polyps, and adenomatous polyps	Hamartomatous polyps	Adenomatous polyps	Hamartomatous polyps	Hamartomas polyps (65.8%), juvenile polyps, ganglioneuromas, adenomatous polyps, inflammatory polyps, leiomyomas, lipomas, lymphoid polyps, and hyperplastic polyps

### 3.6. Treatment

Currently, there are no specific therapeutic options for CCS, and glucocorticoids are mainly used in the clinic; their therapeutic mechanisms may involve reducing gastrointestinal inflammation and suppressing autoimmune responses. The reported initial doses vary, ranging from 30 to 80 mg/d.^[31,32]^ In contrast, Watanabe C found that the optimal dose of glucocorticosteroids is 30 to 49 mg/d.^[8]^ Some studies have shown that some cases have shown significant improvement in symptoms and endoscopic polyp performance after glucocorticosteroid intervention combined with drugs such as 5-aminosalicylic acid, immunosuppressants,^[33]^ and antitumor necrosis factor-alpha. Other drug interventions resulted in significant improvement in symptoms and the endoscopic presentation of polyps. Schulte’s study reported complete regression of all intestinal polyps in a patient with CCS treated with glucocorticosteroids in combination with mesalazine, with no recurrence for 14 years.^[34]^ Langevin’s study found that a patient with CCS treated with glucocorticosteroids and immunosuppressants for 2 years had no recurrence of intestinal polyps.^[35]^ Treatment with corticosteroids and immunosuppressants for 2 weeks resulted in significant symptomatic relief. A follow-up endoscopy 8 months later showed improvement in the number and size of polyps. For refractory cases with poor response to conventional treatment regimens, evidence from clinical studies suggests that immunomodulatory therapies (e.g., tacrolimus) and biologics (including TNF-α antagonists) have potential therapeutic value.^[35,36]^ Roham Salman Roghani^[37]^ attempted to taper hormone therapy in a patient with CCS. The patient experienced a recurrence of diarrheal symptoms and was later switched to treatment with azathioprine in combination with infliximab, which ultimately resulted in successful discontinuation of the glucocorticosteroid. A follow-up endoscopy 24 months later revealed that the polyps had significantly regressed. In addition, for *H pylori*-positive patients with CCS, eradication therapy can effectively alleviate the condition.^[15]^ Meanwhile, for patients with comorbid anxiety and depressive states, anxiolytic and antidepressant treatments have also been shown to contribute to an improvement in symptoms.^[38]^ Other treatments reported in the literature include traditional Chinese medicine,^[39]^ nonsteroidal anti-inflammatory drugs,^[40]^ and surgery.^[41]^ For gastrointestinal polyps, endoscopic resection can also be performed to prevent the malignant transformation of polyps. In our case, the patient’s gastrointestinal symptoms were significantly relieved in the short term after the use of glucocorticosteroids combined with mesalazine, as well as some symptomatic supportive treatments. However, due to the patient’s self-discontinuation of medication, we were unable to observe any changes in the ectodermal and endoscopic gastrointestinal polyps. This highlights the importance of patient adherence to the treatment of CCS.

### 3.7. Summary

CCS is a rare and clinically diverse disease. Due to limited awareness of the disease among clinicians, CCS is often misdiagnosed or underdiagnosed. This case, which initially manifested as chronic diarrhea and was misdiagnosed as acute enteritis, illustrates the diagnostic challenges associated with CCS. Furthermore, our report demonstrates the marked improvement in gastrointestinal symptoms following treatment with glucocorticoids combined with mesalazine, supporting the potential efficacy of this regimen in CCS management. We underscore the essential role of a multidisciplinary therapeutic approach, encompassing nutritional support, electrolyte repletion, endoscopic surveillance, and psychological intervention. Clinicians should maintain a high index of suspicion for CCS in patients presenting with chronic diarrhea, ectodermal changes, and diffuse gastrointestinal polyps, especially when common causes are excluded, and total gastrointestinal endoscopy and tissue biopsy are recommended. Early recognition allows timely initiation of corticosteroid-based therapy and supportive treatment, improving prognosis and reducing the risk of complications. Incorporating molecular insights may further guide personalized management strategies in the future.

This single-center case report highlights 3 key limitations in current CCS research: the low evidence level of single cases, insufficient sample sizes due to the disease’s rarity, and the lack of control groups. Future research should focus on establishing an international multicenter registry system to strengthen the theoretical foundation for improving the prevention and treatment system for rare diseases.

## Author contributions

**Conceptualization:** Yufang Wang.

**Data curation:** Junjun Wu, Yufang Wang.

**Formal analysis:** Shenlu Wu.

**Supervision:** Yufang Wang.

**Writing – original draft:** Shenlu Wu.

**Writing – review & editing:** Junjun Wu, Yufang Wang.

## References

[1] Cronkhite LW Jr. Canada WJ. Generalized gastrointestinal polyposis; an unusual syndrome of polyposis, pigmentation, alopecia and onychotrophia. N Engl J Med. 1955;252:1011–5. 14383952 10.1056/NEJM195506162522401

[2] Jarnum S Jensen H. Diffuse gastrointestinal polyposis with ectodermal changes. A case with severe malabsorption and enteric loss of plasma proteins and electrolytes. Gastroenterology. 1966;50:107–18. 5900949

[3] Daniel ES Ludwig SL Lewin KJ Ruprecht RM Rajacich GM Schwabe AD. The Cronkhite-Canada Syndrome. An analysis of clinical and pathologic features and therapy in 55 patients. Medicine (Baltimore). 1982;61:293–309. 7109958

[4] Liu S You Y Ruan G. The long-term clinical and endoscopic outcomes of Cronkhite-Canada syndrome. Clin Transl Gastroenterol. 2020;11:e00167. 32352683 10.14309/ctg.0000000000000167 PMC7263663

[5] Wu ZY Sang LX Chang B. Cronkhite-Canada syndrome: from clinical features to treatment. Gastroenterology report. 2020;8:333–42. 33163187 10.1093/gastro/goaa058 PMC7603875

[6] Wang N Xiang Y Tao L. Cronkhite-Canada syndrome: a case report and literature review. Medicine (Baltimore). 2024;103:e40242. 39470508 10.1097/MD.0000000000040242 PMC11521078

[7] She Q Jiang JX Si XM Tian XY Shi RH Zhang GX. A severe course of Cronkhite-Canada syndrome and the review of clinical features and therapy in 49 Chinese patients. Turk J Gastroenterol. 2013;24:277–85. 24226722 10.4318/tjg.2013.0527

[8] Watanabe C Komoto S Tomita K. Endoscopic and clinical evaluation of treatment and prognosis of Cronkhite-Canada syndrome: a Japanese nationwide survey. J Gastroenterol. 2016;51:327–36. 26216651 10.1007/s00535-015-1107-7 PMC4805704

[9] Slavik T Montgomery EA. Cronkhite–Canada syndrome six decades on: the many faces of an enigmatic disease. J Clin Pathol. 2014;67:891–7. 25004941 10.1136/jclinpath-2014-202488

[10] Ong S Rodriguez-Garcia C Grabczynska S. Alopecia areata incognita in Cronkhite-Canada syndrome. Br J Dermatol. 2017;177:531–4. 28029683 10.1111/bjd.15293

[11] Chuamanochan M Tovanabutra N Mahanupab P Kongkarnka S Chiewchanvit S. Nail matrix pathology in cronkhite-canada syndrome: the first case report. Am J Dermatopathol. 2017;39:860–2. 29058694 10.1097/DAD.0000000000000898

[12] Sweetser S Ahlquist DA Osborn NK. Clinicopathologic features and treatment outcomes in Cronkhite-Canada syndrome: support for autoimmunity. Dig Dis Sci. 2012;57:496–502. 21881972 10.1007/s10620-011-1874-9

[13] Riegert-Johnson DL Osborn N Smyrk T Boardman LA. Cronkhite-Canada syndrome hamartomatous polyps are infiltrated with IgG4 plasma cells. Digestion. 2007;75:96–7. 17510553 10.1159/000102963

[14] Wen XH Wang L Wang YX Qian JM. Cronkhite-Canada syndrome: report of six cases and review of literature. World J Gastroenterol. 2014;20:7518–22. 24966624 10.3748/wjg.v20.i23.7518 PMC4064099

[15] Okamoto K Isomoto H Shikuwa S Nishiyama H Ito M Kohno S. A case of Cronkhite-Canada syndrome: remission after treatment with anti-Helicobacter pylori regimen. Digestion. 2008;78:82–7. 18948692 10.1159/000165354

[16] Bandyopadhyay D Hajra A Ganesan V. Cronkhite-Canada syndrome: a rare cause of chronic diarrhoea in a young man. Case Rep Med. 2016;2016:4210397. 26941798 10.1155/2016/4210397 PMC4749779

[17] Dawra S Sharma V Dutta U. Clinical and endoscopic remission in a patient with Cronkhite-Canada Syndrome. Clin Gastroenterol Hepatol. 2018;16:e84–5. 29627427 10.1016/j.cgh.2017.09.023

[18] Goto A. Cronkhite-Canada syndrome: epidemiological study of 110 cases reported in Japan. Nihon Geka Hokan. 1995;64:3–14. 8534187

[19] Murata I Yoshikawa I Endo M. Cronkhite-Canada syndrome: report of two cases. J Gastroenterol. 2000;35:706–11. 11023043 10.1007/s005350070051

[20] Boland BS Bagi P Valasek MA. Cronkhite Canada Syndrome: significant response to infliximab and a possible clue to pathogenesis. Am J Gastroenterol. 2016;111:746–8. 10.1038/ajg.2016.92 PMC5824983 27151126

[21] Zhu LP Zhong WL Wang ZG. Cronkhite-Canada syndrome: an investigation in clinical features and pathogenesis. J Dig Dis. 2021;22:663–71. 34697888 10.1111/1751-2980.13062

[22] Ho V Banney L Falhammar H. Hyperpigmentation, nail dystrophy and alopecia with generalised intestinal polyposis: Cronkhite-Canada syndrome. Australas J Dermatol. 2008;49:223–5. 18855786 10.1111/j.1440-0960.2008.00474.x

[23] Poplaski V Bomidi C Kambal A. Human intestinal organoids from Cronkhite-Canada syndrome patients reveal link between serotonin and proliferation. J Clin Invest. 2023;133:e166884. 37909332 10.1172/JCI166884 PMC10617781

[24] Liu S Zhi Y Zhang R. Cronkhite–Canada syndrome as inflammatory hamartomatous polyposis: new evidence from whole transcriptome sequencing of colonic polyps. Orphanet J Rare Dis. 2024;19:35. 38297356 10.1186/s13023-024-03038-8 PMC10832113

[25] Lu Y Huang F Wang Y Zhou J Zhao Q Liu L. Clinical and Endoscopic Characteristics of Chinese Cronkhite-Canada syndrome patients: a retrospective study of 103 Cases. Dig Dis. 2021;39:488–95. 33440392 10.1159/000514354

[26] Flannery CM Lunn JA. Cronkhite-Canada Syndrome: an unusual finding of gastro-intestinal adenomatous polyps in a syndrome characterized by hamartomatous polyps. Gastroenterology report. 2015;3:254–7. 24982130 10.1093/gastro/gou041 PMC4527259

[27] De Petris G Chen L Pasha SF Ruff KC. Cronkhite-Canada syndrome diagnosis in the absence of gastrointestinal polyps: a case report. Int J Surg Pathol. 2013;21:627–31. 23515557 10.1177/1066896913480832

[28] Bettington M Brown IS Kumarasinghe MP de Boer B Bettington A Rosty C. The challenging diagnosis of Cronkhite-Canada syndrome in the upper gastrointestinal tract: a series of 7 cases with clinical follow-up. Am J Surg Pathol. 2014;38:215–23. 24418855 10.1097/PAS.0000000000000098

[29] Ueyama H Fu KI Ogura K Murata S Miyazaki A. Successful treatment for Cronkhite-Canada syndrome with endoscopic mucosal resection and salazosulfapyridine. Tech Coloproctol. 2014;18:503–7. 22847839 10.1007/s10151-012-0863-0

[30] Fan RY Wang XW Xue LJ An R Sheng JQ. Cronkhite-Canada syndrome polyps infiltrated with IgG4-positive plasma cells. World J Clin Cases. 2016;4:248–52. 27574615 10.12998/wjcc.v4.i8.248 PMC4983698

[31] Tang YC. Cronkhite-Canada syndrome with esophagus involvement and six-year follow-up: a case report. World J Gastroenterol. 2024;30:984–90. 38516236 10.3748/wjg.v30.i8.984 PMC10950646

[32] Pham JT Kisiel JB Sweetser S. Cronkhite-Canada syndrome: treatment responses and improved overall survival. Int J Colorectal Dis. 2023;38:39. 36781513 10.1007/s00384-023-04332-w

[33] Iqbal U Chaudhary A Karim MA Anwar H Merrell N. Cronkhite-Canada syndrome: a rare cause of chronic diarrhea. Gastroenterology Res. 2017;10:196–8. 28725309 10.14740/gr820w PMC5505287

[34] Schulte S Kütting F Mertens J. Case report of patient with a Cronkhite-Canada syndrome: sustained remission after treatment with corticosteroids and mesalazine. BMC Gastroenterol. 2019;19:36. 30813906 10.1186/s12876-019-0944-x PMC6391814

[35] Langevin C Chapdelaine H Picard JM Poitras P Leduc R. Sirolimus in refractory Cronkhite-Canada syndrome and focus on standard treatment. J Invest Med High Impact Case Rep. 2018;6:2324709618765893. 10.1177/2324709618765893 PMC5871038 29619395

[36] Watanabe D Ooi M Hoshi N. Successful treatment of Cronkhite-Canada syndrome using anti-tumor necrosis factor antibody therapy. Endoscopy. 2014;46(Suppl 1 UCTN):E476–477. 25314205 10.1055/s-0034-1377539

[37] Salman Roghani R De Castro J Ajumobi AB. Clinical and endoscopic response to anti-tumor necrosis factor-alpha antibody therapy in a patient with Cronkhite-Canada Syndrome. J Invest Med High Impact Case Rep. 2023;11:23247096231179451. 10.1177/23247096231179451 PMC10265369 37278538

[38] Dore MP Satta R Murino A Pes GM. Long-lasting remission in a case of Cronkhite-Canada syndrome. BMJ Case Rep. 2018;2018:bcr2017223527. 10.1136/bcr-2017-223527 PMC5950697 29739761

[39] Hu H Wu Y Zhang Y Zhang L Zhang J Zhang R. Comprehensive treatment of Cronkhite-Canada syndrome: a case report and literature review. Medicine (Baltimore). 2023;102:e32714. 36820546 10.1097/MD.0000000000032714 PMC9907941

[40] Chakrabarti S. Cronkhite-Canada Syndrome (CCS)-a rare case report. J Clin Diagn Res. 2015;9:OD08–09. 10.7860/JCDR/2015/11919.5700 PMC4413105 25954656

[41] Samalavicius NE Lunevicius R Klimovskij M Kildušis E Zažeckis H. Subtotal colectomy for severe protein-losing enteropathy associated with Cronkhite-Canada syndrome: a case report. Colorectal Dis. 2013;15:e164–165. 23323676 10.1111/codi.12111

